# *Apocynum venetum* leaf extract alleviated doxorubicin-induced cardiotoxicity by regulating organic acid metabolism in gut microbiota

**DOI:** 10.3389/fphar.2023.1286210

**Published:** 2023-11-23

**Authors:** Zhenxiong Zhao, Shenglu Jiang, Qing Fan, Kuo Xu, Yubin Xu, Feiqiang Wu, Xihong Zhang, Ting Wang, Zhelin Xia

**Affiliations:** ^1^ Taizhou Central Hospital (Taizhou University Hospital), Taizhou, Zhejiang, China; ^2^ School of Medicine, Taizhou University, Taizhou, Zhejiang, China; ^3^ Shandong Cancer Hospital and Institute, Shandong First Medical University and Shandong Academy of Medical Sciences, Jinan, China; ^4^ Marine Traditional Chinese Medicine Research Center, Shandong University of Traditional Chinese Medicine, Jinan, China; ^5^ Taizhou Hospital of Zhejiang Province Affiliated to Wenzhou Medical University, Taizhou, Zhejiang, China

**Keywords:** gut microbiota, *Apocynum venetum* leaf, doxorubicin, cardiotoxicity, indole-3-propionic acid

## Abstract

*Apocynum venetum* leaf is commonly utilized for its beneficial effects in reducing blood pressure, inducing sedation, promoting diuresis, anti-aging, and cardioprotection, which also exhibit positive effects on the gut microbiota. The gut microbiota plays a role as an endocrine organ by producing bioactive metabolites that can directly or indirectly impact host physiology, specifically cardiovascular diseases. In this study, main chemical components of *A. venetum* leaf extract (AVLE) were identified by LC-MS, and an orally administered AVLE was employed to treat mice with doxorubicin (Dox)-induced cardiotoxicity. The results showed that AVLE contained hyperoside and oganic acids. The pharmacological findings revealed that AVLE regulated the gut microbiota, resulting in a significant increase in the levels of two organic acids, indole-3-propionic acid (IPA) and acetic acid (AA). Both IPA and AA exhibited the ability to reduce BNP, CK, and LDH levels in mice with Dox-induced cardiotoxicity. Moreover, IPA demonstrated an improvement in Dox-induced cardiac injury by inhibiting apoptosis, while AA promoted increased secretion of ghrelin through the parasympathetic nervous system, subsequently reducing cardiac fibrosis by decreasing collagen I, collagen III, and activin A. Hence, our study demonstrates that AVLE exerts a beneficial cardioprotective effect by modulating the gut microbiota, providing a potential novel target for the treatment and prevention of Dox-induced cardiotoxicity.

## 1 Introduction

*Apocynum venetum*, commonly known as “Luobuma” in Chinese and “Rafuma” in Japanese, is a perennial herbaceous shrub that is widely spread across the temperate regions of Asia, Europe, and North America (Xiang et al., 2023). The bioactive components of *A. venetum* primarily include flavonoids and polysaccharides, which are both bioactive components with low bioavailability (Gao et al., 2021). *A. venetum* extract possesses various biological effects, such as lowering blood pressure levels, sedation, diuresis, anti-aging, and improving immunity (Xiang et al., 2023). According to recent studies, *A. venetum* extract exhibits good cardioprotective effects through the AKT/Bcl-2 signalling pathway (Zhang et al., 2022). However, the absorption of flavonoids and polysaccharides, the main bioactive components of A. venetum, in the gut is challenging, which hinders the elucidation of the cardioprotective mechanism. *A. venetum* leaf extracts (AVLEs) have been shown to modulate the gut microbiota, resulting in beneficial effects on gut microecology. This finding opens up new avenues for investigating the underlying mechanisms (Yuan et al., 2020).

The utilization of doxorubicin (Dox), an anthracycline chemotherapy agent, in the treatment of tumors has been shown to significantly enhance remission rates and overall survival in cancer patients (Rivankar, 2014; Li et al., 2022; Sheibani et al., 2022). However, the therapeutic application of anthracyclines is hindered by their propensity to induce dose-dependent cardiotoxicity, leading to the development of heart failure and mortality. Particularly, Dox-induced cardiotoxicity, characterized by left ventricular dysfunction and heart failure, represents its most severe manifestation (Lu et al., 2022; Magdy et al., 2022; Ozcan et al., 2023). Despite its notable cardiotoxic effects, Dox remains the primary chemotherapy drug for breast cancer, lung cancer, lymphoma, and acute leukemia, thereby resulting in irreversible cardiac damage among cancer patients. Consequently, the identification of effective drugs capable of mitigating Dox-induced cardiotoxicity is of utmost importance. Current research indicates that Dox exerts its cardiotoxic effects through various mechanisms, including autophagy, ferroptosis, necroptosis, fibrosis, and apoptosis (Timm and Tyler, 2020; Christidi and Brunham, 2021). Nevertheless, the precise mechanisms underlying Dox-induced cardiomyocyte death remain elusive, despite extensive investigations.

Our understanding of the impact of gut microbiota on human health and diseases has significantly expanded, shedding light on the influence of microbial composition and function on the human host (Zhao et al., 2018; Zhao et al., 2022). According to recent studies, the gut microbiota functions like an endocrine organ by generating bioactive metabolites that can directly or indirectly impact host physiology (Wang et al., 2021), particularly cardiovascular disease (Kazemian et al., 2020; Anselmi et al., 2021). Notably, newly discovered gut microbial metabolic pathways, such as the production of trimethylamine/trimethylamine N-oxide and secondary bile acids, play a role in the development and progression of cardiovascular diseases, including heart failure. (Tang et al., 2019; Zhang et al., 2021). Organic acids generated by the gut microbiota, including short-chain fatty acids (SCFAs) and amino acid metabolites, have also been implicated in cardiovascular disease (Bartolomaeus et al., 2019; Xue et al., 2022). Recent studies have mechanistically linked SCFAs to the development of hypertension through specific host receptor recognition, including Gpr41, Gpr43, and Olfr78, leading to alterations in blood pressure (Pluznick, 2014). Furthermore, preclinical studies have identified acetate-producing bacteria as a potential protective intervention for hypertension, adverse cardiac hypertrophy, and fibrosis development (Marques et al., 2017). Indole-3-acetic acid (IAA) and indole-3-propionic acid (IPA) are tryptophan metabolites in gut microbiota. IAA is an independent predictor of mortality and cardiovascular events in patients with chronic kidney disease, which induces endothelial inflammation and oxidative stress and activates an inflammatory AhR/p38MAPK/NF-κB pathway (Dou et al., 2015). Dietary IPA supplementation alleviates atherosclerotic plaque development in ApoE−/− mice by facilitating macrophage reverse cholesterol transport (Xue et al., 2022). Gut microbiota can interact with flavonoids, in one direction to produce new flavonoid-derived compounds with better bioavailability, in another direction to produce gut microbiota-derived compounds such as SCFAs (Cheng et al., 2023). In addition, herbal medicine extract exhibited good antifungal and antiviral activity (Bagheri et al., 2020). Therefore, targeting the endogenous metabolites produced by the gut microbiota holds promise for the prevention and treatment of cardiovascular diseases.

In this study, we focused on gut microbiota regulation of *A. venetum* leaf extracts on Dox-induced cardiotoxicity. Echocardiography was performed to observe the therapeutic of *A. venetum*. Organic acids generated by gut microbiota were examined to explore the effects of *A. venetum* extract on gut microbiota. Consequently, the mechanisms for cardioprotection of AA and IPA were partially clarified. The findings of this study may provide a theoretical basis for the treatment/prevention of Dox-induced cardiotoxicity and can aid in the search of its potential cardioprotective functions in gut microbiota.

## 2 Materials and methods

### 2.1 Materials and AVLE preparation

To obtain AVLE, the following procedure was conducted (Wu et al., 2018): Firstly, 100 g of *A. venetum* leaves were refluxed in a mixture of aqueous ethanol (70%, v/v) and 60 mL with 1 h. This process was repeated twice. The combined alcoholic extract was then evaporated to obtain a dry extract weighing 20.1 g. Next, 10 g of the extract was dissolved in 200 mL of hot water and the pH was adjusted to 3.0 using sulfuric acid. The solution was filtered, and the filtrate was subjected to chromatography on a Diaion HP-20 column with a diameter of 3.6 cm and a height of 18 cm, provided by Sigma-Aldrich in St. Louis, MO, United States. The column was eluted with 200 mL of water followed by 200 mL of aqueous ethanol (70%, v/v). The fraction collected from the aqueous ethanol elution was evaporated to obtain AVLE with a yield of 3.1 g. And the main components were identified by Q-Exactive HF (Thermo Fisher Scientific). Doxorubicin (purity >99%) was got from Rhawn (Shanghai, China). Acetic acid (AA), propionic acid (PA), 3-indole propionic acid (IPA), butyric acid (BA), valeric acid (VA), isovaleric acid (iVA), hexanoic acid (HA) and isohexanoic acid (iHA) were obtained from J&K Scientific (Beijing, China) with the purities of >99%. 2-Bromoacetophenone (purity >98%), propidium iodide (purity >98%) and Ghrelin (Gh, purity >95%) were got from Aladdin (Shanghai, China). The Assay Kit of brain natriuretic peptide (BNP), creatine kinase (CK), lactate dehydrogenase (LDH) and Ghrelin (GH) were bought from Nanjing Jiancheng Bioengineering Institute (Nanjing, China).

### 2.2 Animals

Male C57BL/6 mice weighing 18–20 g were purchased from Zhejiang Vital River Company (Hangzhou, China), were kept under 12 h light/dark cycles, and allowed free access to food and water. All the mice have adapted to the environment for 1 week before the experiment started. The experiment was carried out in strict accordance with the ethical guidelines for experimental animals, and was approved by the Animal Ethics Committee of the Experimental Animal Institute of Taizhou University (No. TZXY-2022-20221052).

### 2.3 AVLE alleviated doxorubicin-induced cardiotoxicity

Thirty male C57BL/6 mice were randomly divided into five groups, and the group information was as follows (Ma et al., 2012): Group 1, normal group (treated with saline); Group 2, Dox group (model group), (treated with Dox 4 mg/kg per week for 5 weeks, intraperitoneally (i.p.)); Group 3, pseudo germ-free (PGF) group, which was orally treated with AVLE (200 mg/kg/d) and broad-spectrum antibiotics (cefadroxil 100 mg/kg/d, terramycin 300 mg/kg/d and erythromycin 300 mg/kg/d, every 3 days during the treatment process) and Dox 4 mg/kg per week for 5 weeks; Group 4, A1 group (treated with AVLE 100 mg/kg/d, orally); Group 5, A2 group (treated with AVLE 200 mg/kg/d, orally). Group 3, 4 and 5 were also treated with Dox 4 mg/kg per week for 5 weeks, intraperitoneally and treated with AVLE 1 week before the injection of Dox, and treatment was continued until 1 week after the last Dox injection. After 7 weeks of AVLE treatment, mice cardiac function was determined via echocardiography. Subsequently, serum, faeces, and heart were collected after the mice were anesthetized. The hearts were taken out and were fixed in 4% tissue cell fixative (4% paraformaldehyde) for 24 h, dehydrated by an automatic dehydrator for 16 h, and then routinely embedded in a paraffin embedding machine for preparation of heart tissue sections. H&E and Masson staining were performed according to the reported methods.

### 2.4 Echocardiography

Transthoracic ultrasound imaging was conducted on six mice in each group at the Cardiovascular Assessment Facility. The imaging was performed using the Mylab SigmaVet ultrasound machine connected to the SL3116 probe (Esaote, Genoa, Italy). Standard views were used to measure the diameter of the left ventricle at end-diastole and end-systole (referred to as LVEDD and LVESD, respectively). From these measurements, the stroke volume, ejection fraction, and cardiac output of the left ventricle were calculated. Additionally, Doppler recordings were taken to measure the E-wave and A-wave of the mitral valve from a four-chamber view of the left ventricle. Tissue Doppler velocity was assessed at the lateral annulus of the mitral valve (referred to as lateralE0). Throughout the procedure, the mice were given mild anesthesia using 1%–1.5% Isoflurane.

### 2.5 Detection of BNP, CK, LDH and Gh

Serum BNP, CK, LDH and Gh were detected by Assay Kits according to standard procedure of Assay Kit (Nanjing Jiancheng Bioengineering Institute).

### 2.6 Determination of eight organic acids

Eighteen C57BL/6 mice were divided into three groups. Group A1 were orally treated with AVLE (100 mg/kg), and Group A2 were orally treated with AVLE (200 mg/kg). The control group were treated with saline. After 24 h AVLE treatment, the serum were collected from mice fundus venous plexus for organic acids detection.

For detection method, fecal samples were pretreated by dilution (1:1,000, W/V) in water. A 50 μL of serum, fecal sample, or gut microbiota culture medium was mixed with 50 μL of saturated sodium carbonate solution in a 1.5 mL EP tube. The mixture was then vortexed for 15 s and evaporated at 40 °C under a nitrogen stream until dry. Subsequently, 150 μL of a 2-bromoacetophenone solution (10 mg/mL) in acetonitrile was added and incubated at 40 °C for 20 min. After derivatization, the solution was centrifuged at 13,000 g for 5 min. A 100 μL aliquot of the supernatant was taken for analysis. The analysis was performed using liquid chromatography with tandem mass spectrometry (LCMS/MS 8050, Shimadzu Corporation, Kyoto, Japan) equipped with an electrospray ionization (ESI) source. LC separation was carried out using an xBridge C18 column (100 mm × 2.1 mm × 3.5 μm, Waters, Milford, United States) with a flow rate of 0.4 mL/min at 30 °C. The mobile phase consisted of formic acid:water (0.1:100, v/v) (as phase A) and methanol (as phase B), with a binary gradient elution as follows (A:B): 0.01 min: 85:15; 2.00 min: 85:15; 2.01 min: 60:40; 5.00 min: 60:40; 20.00 min: 52:48; 20.01 min: 40:60; 25.00 min: 35:65; 25.01 min: 85:15; 27.00 min: controller stop.

### 2.7 Cell culture

HL-1 cell line (mouse cardiomyocytes) was purchased from Haixing Biosciences (Suzhou, China). HL-1 cells were cultured in Dulbecco’s modified Eagle Medium (DMEM) containing 10% foetal bovine serum (FBS) at 37°C and 5% carbon dioxide (CO2), and the cells were passaged at 70%–80% confluence. For the flow cytometry experiment, the HL-1 cells were pretreated with 5 μM of IPA for 24 h and then treated with 5 μM of Dox for 12 h in DMEM containing 1% FBS.

### 2.8 IPA and AA alleviation doxorubicin-induced cardiotoxicity

Twenty-four C57BL/6 mice were randomly divided into four groups as follows: Group 1, normal group (treated with saline); Group 2, Dox group (model group), (treated with Dox 4 mg/kg per week for 5 weeks, i. p.); Group 3, IPA group (treated with IPA 20 mg/kg/d, orally and Dox 4 mg/kg per week for 5 weeks, i. p.); and Group 4, AA group (treated with sodium acetate 500 mg/kg/d, orally and Dox 4 mg/kg per week for 5 weeks, i. p.). Groups 3 and 4 were treated with IPA or sodium acetate 1 week prior to Dox administration, and treatment was continued until 1 week after the last Dox injection. After 7 weeks of IPA or sodium acetate treatment, mice serum were collected and serum BNP, CK, and LDH were detected using Assay Kits according to standard procedure of Assay Kit.

### 2.9 Flow cytometry analysis

To investigate cell apoptosis, we used the Annexin V-FITC apoptosis kit (Beyotime, Shanghai, China) following these steps. After treatment with Dox in section 2.7, HL-1 cells were collected using 0.25% trypsin and washed twice with cold PBS. The collected cells (0.5–1×10^6^) were then centrifuged at 1,000 g for 5 min and resuspended in 195 μL of Annexin V-FITC solution. Following that, 5 μL of Annexin V-FITC was added and the mixture was kept at 4 °C in the dark for 15 min. Next, 5 μL of Propidium iodide staining solution was added to the tubes and they were placed at 4 °C in the dark for another 15 min. Finally, we used a flow cytometer (Beckman CytoFLEX, Indianapolis, United States) to quantify the apoptotic cell population.

### 2.10 Assessment of AA effect on Gh secretion

Furthermore, we explored the cardioprotective effects of AA by stimulating Gh secretion. Forty-two male C57BL/6 mice were randomly divided into seven groups as follows: Normal Group, which was used as control group; Model Group, which was treated with single-dose Dox 15 mg/kg i. p.; AA Group, which was orally treated with AA 200 mg/kg/d for 3 days; Vagotomy Group, whose dorsal and ventral branches of the vagus nerve were tied using the reported methods (Williams et al., 2003); Vagotomy AA Group, which was orally treated with AA 200 mg/kg/d for 3 days after vagotomy; Sham group, whose nerve was exposed but not tied; and Sham AA group, whose nerve was exposed but not tied and treated with AA 200 mg/kg/d for 3 days. After the 3 days of AA treatment, serum samples were taken out for Gh and BNP detection.

HL-1 cells were pretreated with Gh (50 μM and 100 μM, respectively) for 24 h and then treated with 5 μM of Dox for 12 h in DMEM containing 1% FBS. The mRNA of activin A (Act A), follistatin (FS), collagen I, collagen III were measured by real-time quantitative PCR. Briefly, total RNA from cultured CFs was extracted with the TRIzol reagent, and stored at −80°C. Primers were synthesized by Shyilaibo Co., LTD. (Shanghai, China), as shown in Supplementary Table S1. Fluorescence quantitative RT-PCR was performed according to kit instructions, using the Hieff^®^ qPCR SYBR Green Master Mix and the ABI Prism 7,500 (Applied Biosystems) instrument. The PCR program was set as follows: 95°C for 5 min for one cycle; 95°C for 10 s, 55°C–60°C for 20 s and 72°C for 20 s (for 40 cycles. For data analysis, the comparative threshold cycle (CT) value for Gapdh was used to normalise the loading variations in the real-time PCR reactions. The ΔΔCT value was then obtained by subtracting the control ΔCT values from the corresponding experimental ΔCT values. The ΔΔCT values were converted into a fold difference that was compared to the control using Equation 2^−ΔΔCT^.

### 2.11 Microbial diversity analysis

16S rRNA genes were amplified using specific primers targeting the 16S V3-V4 regions named 340F-805R. The methods were the same as those used in our previous study (Zhao et al., 2018). Operational taxonomic units (OTUs) with similarity over 97% were selected for taxonomy identification with Greengenes database (V.13.8).

### 2.12 Statistical analysis

Statistical analyses were conducted using Student’s t-test with GraphPad Prism Version 8 (GraphPad Software, CA, United States). Data are expressed as mean ± standard deviation, and *p* values less than 0.05 were considered statistically significant.

## 3 Results

### 3.1 Main active ingredients of AVLE

The main active ingredients of AVLE was identified by Q-Exactive HF LC-MS system. The total ion chromatogram (TIC) in the positive and negative ion modes are shown in Supplementary Figure S1. A total of 214 compounds were identified and the top 20 active ingredients were shown in Supplementary Table S2. The main chemical compositions were hyperoside and oganic acids.

### 3.2 AVLE alleviated doxorubicin-induced cardiotoxicity

**FIGURE 1:**
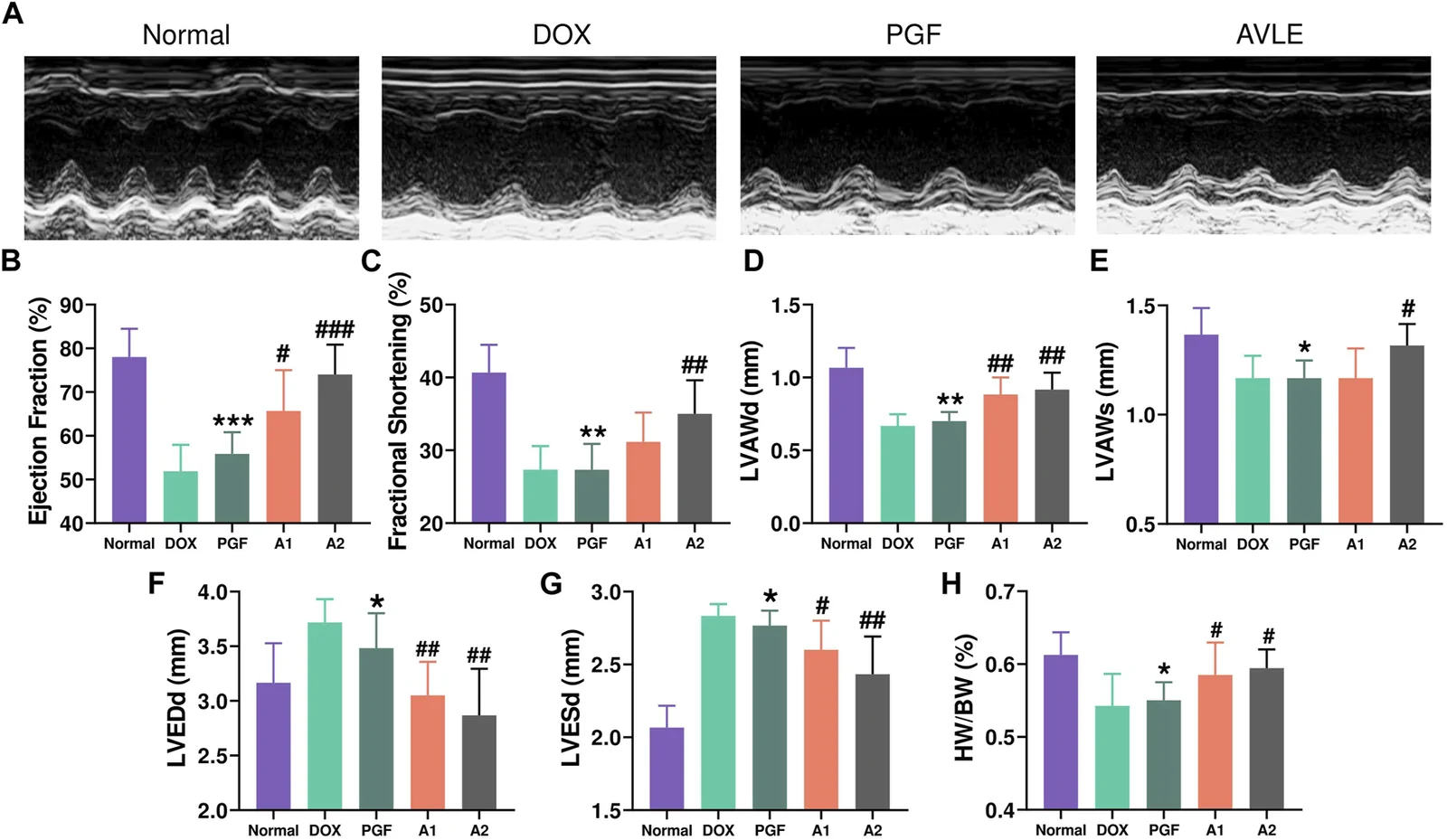
Effects of AVLE on cardiac function after 5-week Dox treatment in mice. **(A)** Representative images of echocardiography **(B)** the ejection fraction (EF%) of mice in different groups **(C)** the fractional shortening (FS%) **(D)** left ventricular anterior diastolic wall thickness (LVAWd) **(E)** left ventricular anterior systolic wall thickness (LVAWs) **(F)** left ventricular end diastolic diameter (LVEDd) **(G)** left ventricular end systolic diameter (LVESd) **(H)** the ratios of heart weights to body weights were calculated. Data are presented as mean ± SD; n = 6, ^#^ *p* < 0.05, ^##^ *p* < 0.01, ^###^ *p* < 0.001 compared with model group; *p < 0.05, ^**^ *p* < 0.01, ^***^ *p* < 0.001 compared with A2 group.

**FIGURE 2:**
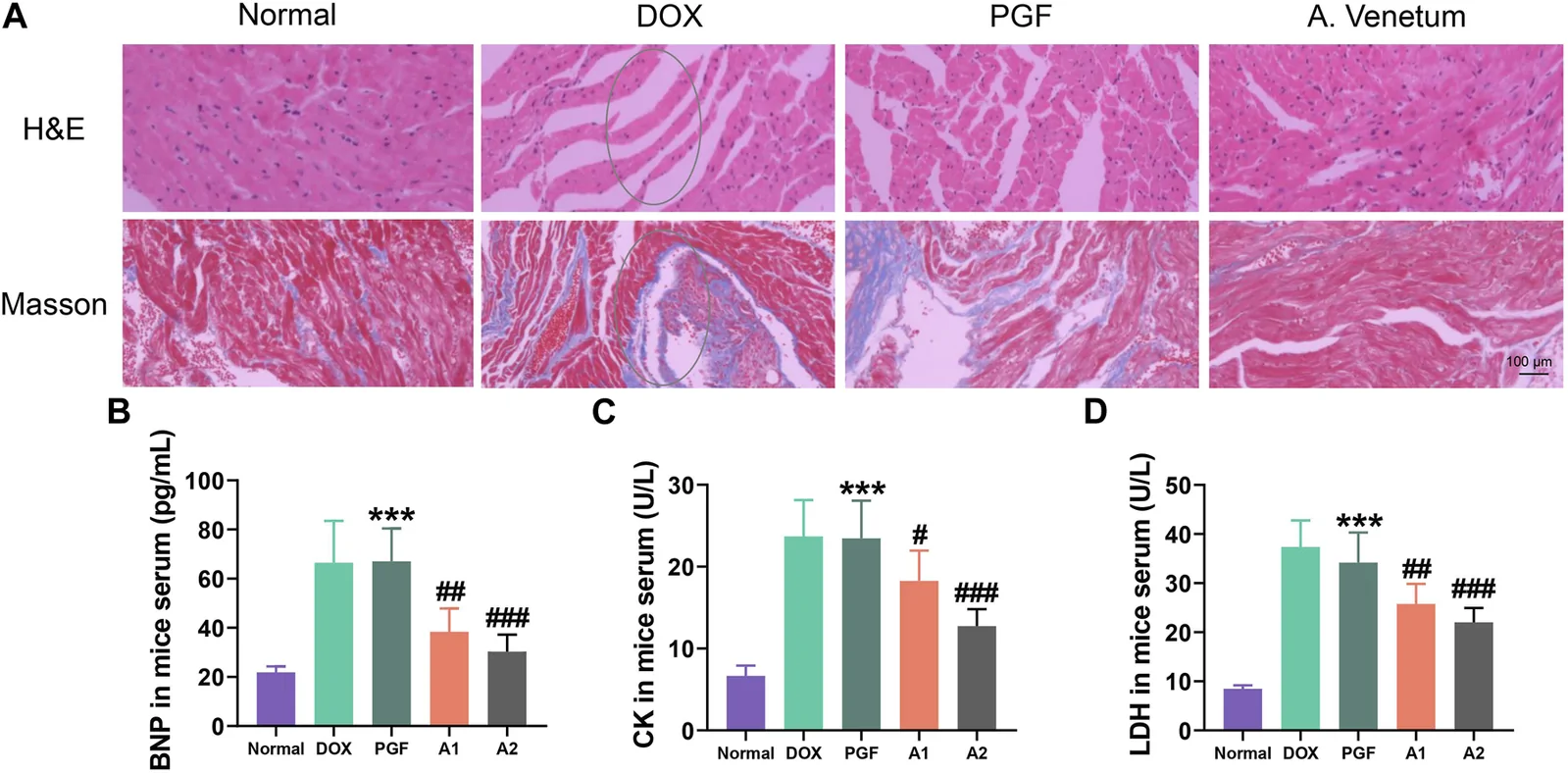
Cardiac tissue staining and detection of cardiac injury indicators. **(A)** H&E and Masson staining of cardiac tissue after AVLE treatment **(B–D)** Serum brain natriuretic peptide (BNP), creatine kinase (CK) and lactate dehydrogenase (LDH) of Dox-induced cardiotoxicity mice after AVLE treatment. Data are presented as mean ± SD; n = 6, ^#^ *p* < 0.05, ^##^ *p* < 0.01, ^###^ *p* < 0.001 compared with model group; ^***^ *p* < 0.001 compared with A2 group.

We established a chronic mouse model of cardiomyopathy by administering Doxorubicin (Dox) through continuous injections at a dosage of 4 mg/kg per week for 5 weeks. Cardiac function was evaluated using echocardiography 1 week after the final Dox injection. The echocardiography results (Figure 1) demonstrated that Dox administration led to cardiac dysfunction in mice. Subsequently, treatment with AVLE resulted in a significant dose-dependent improvement in cardiac function. To further investigate the cardiac protective effects of AVLE, histological analyses including H&E and Masson staining were performed. The H&E and Masson staining results (Figure 2A) revealed that AVLE treatment restored the abnormal cellular structure to normal and reduced myocardial fibrosis. The protective effect of AVLE on heart function was also confirmed by the measurements of BNP, CK, LDH, and additional echocardiography results (Figure 1; Figures 2B–D). However, the group treated with AVLE in combination with PGF did not show alleviation of Dox-induced cardiotoxicity, suggesting that the modulation of gut microbiota may be a crucial target for AVLE in mitigating Dox-induced cardiotoxicity.

### 3.3 AVLE regulated organic acids generated by gut microbiota

**FIGURE 3:**
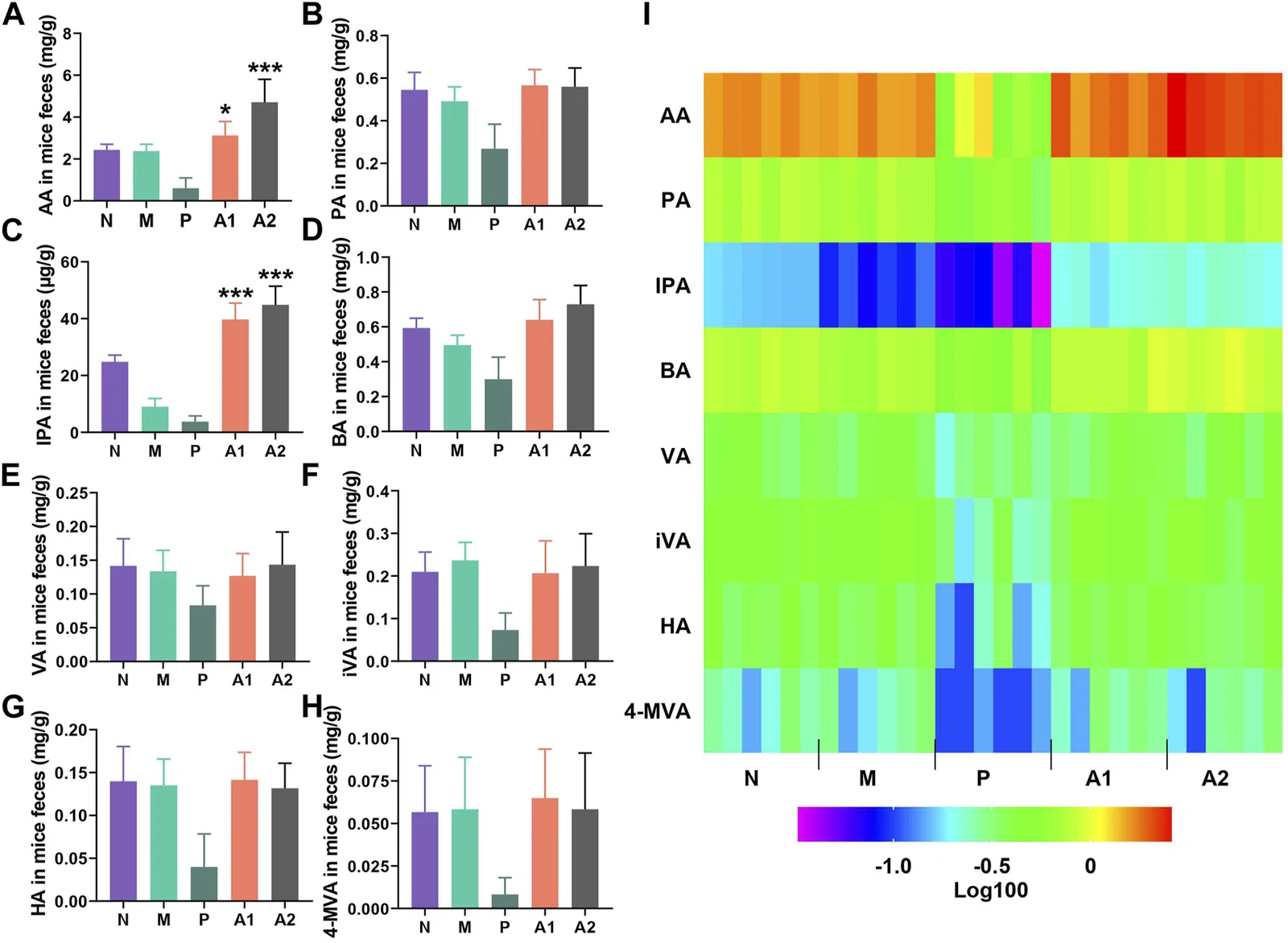
AVLE regulating organic acids generated by gut microbiota. **(A–H)** Detection of acetic acid (AA), propionic acid (PA), indole-3- propionic acid (IPA), butyric acid (BA), valeric acid (VA), isovaleric acid (iVA), hexanoic acid (HA) and 4-methylvaleric acid (4-MVA) in mice feaces after AVLE treatment **(I)** heatmap of organic acids concentration. N, normal group; M, model group; P, PGF group. Data are presented as mean ± SD; n = 6, *p < 0.05, ^***^ *p* < 0.001 compared with model group.

**FIGURE 4:**
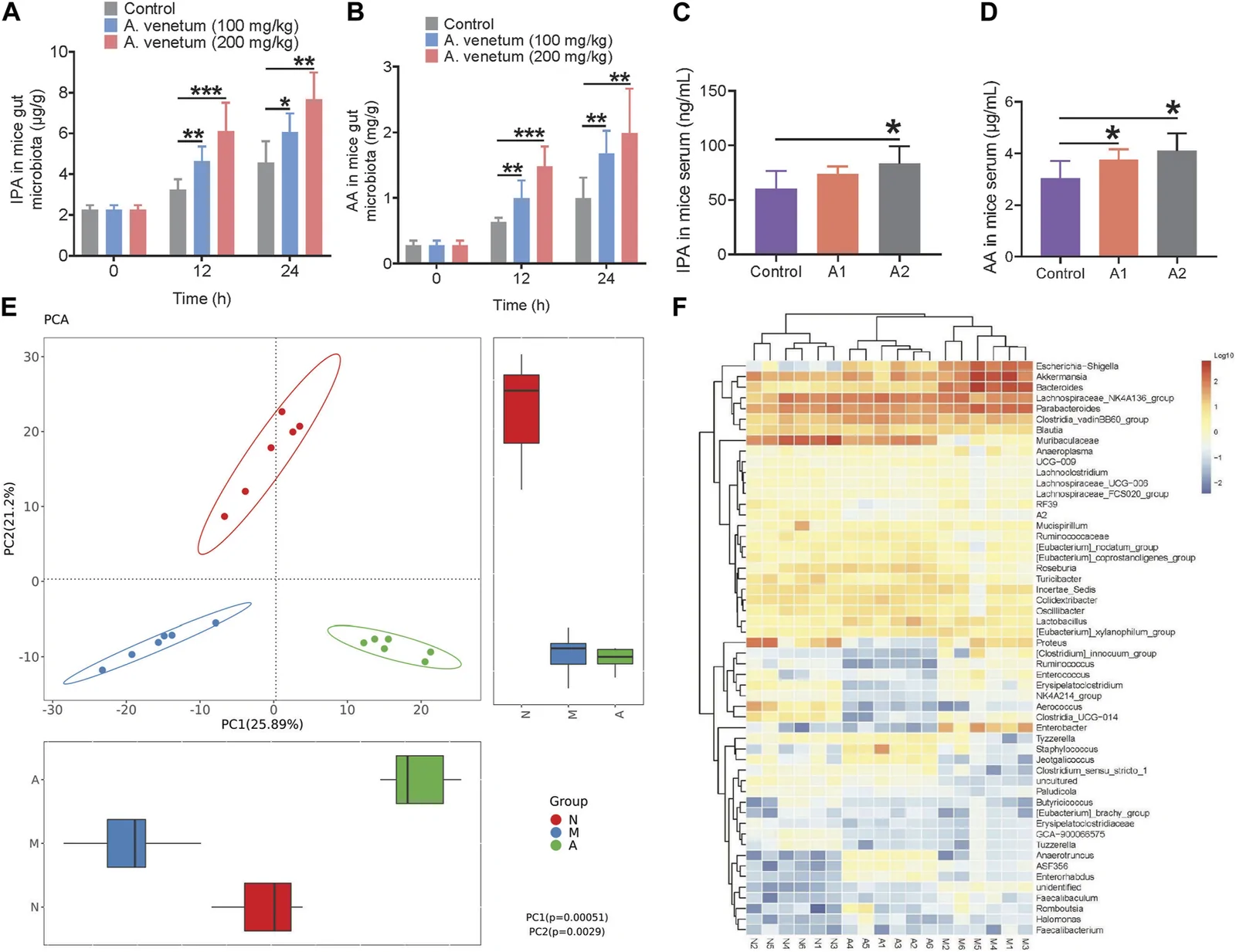
Regulation of AVLE on mice gut microbiota. **(A,B)** The concentration of IPA and AA in gut microbiota culture system *in vitro* **(C,D)** IPA and AA in mice serum after treated with AVLE for 24 h **(E)** principal components analysis (PCA) score plot of three groups, N (normal group), M (model group), A (A2 group) (F) heatmap of top 50 genera in three groups, N (normal group), M (model group), A (A2 group). Data are presented as mean ± SD; n = 6, *p < 0.05, **p < 0.05, ***p < 0.001.

The levels of eight organic acids, namely, acetic acid (AA), propionic acid (PA), indole-3-propionic acid (IPA), butyric acid (BA), valeric acid (VA), isovaleric acid (iVA), hexanoic acid (HA), and 4-methylvaleric acid (4-MVA), were quantified using LC-MS/MS to investigate the regulatory effects of AVLE on gut microbiota-derived organic acids. The results are presented in Figure 3. Notably, the PGF group exhibited the lowest levels of organic acids among the five groups due to the disruption of gut microbiota caused by broad-spectrum antibiotics. In contrast, AVLE treatment stimulated the production of AA and IPA in a dose-dependent manner within the gut microbiota. The heat map in Figure 3I illustrates the alterations in the levels of the eight organic acids influenced by AVLE in the Dox-induced cardiotoxicity mouse model, with IPA and AA significantly increasing following AVLE treatment. To confirm the stimulating effect of AVLE, an *in vitro* gut microbiota culture system was utilized. The results demonstrated a significant dose-dependent increase in IPA and AA levels in the *in vitro* gut microbiota culture system after 12 and 24 h of AVLE treatment (Figures 4A, B). Consistently, the levels of IPA and AA in mouse serum significantly increased after 24 h of AVLE treatment (Figures 4C, D). These findings suggest that AVLE can stimulate the production of AA and IPA within the gut microbiota, leading to increased levels of AA and IPA in the serum.

### 3.4 Bacterial composition modified by AVLE

The gut microbiota composition was analyzed by sequencing the 16S rRNA gene, specifically targeting the V3 and V4 regions, using barcoded pyrosequencing. The results demonstrated significant differences in the gut microbiota among the three groups (Figure 4E and Supplementary Figure S2A). The relative abundance of genera in the gut microbiota of the mice from the three groups is presented in Supplementary Figure S2B, indicating the involvement of AVLE in regulating the Dox-induced gut microbiota imbalance. A heat map displaying the top 50 bacterial genera exhibiting the most substantial changes in abundance after AVLE exposure is depicted in Figure 4F. Out of the 50 genera, the abundance of 11 genera (*Escherichia−Shigella*, *Akkermansia*, *Bacteroides*, *Clostridium*, *Ruminococcus*, *Enterobacter*, *Anaerotruncus*, *Enterorhabdus*, *Faecalibaculum*, *Romboutsia*, and *Halomonas*) increased and the abundance of 10 genera (*Muribaculaceae*, *Lachnoclostridium*, *Ruminococcaceae*, *Eubacterium*, *Roseburia*, *Turicibacter*, *Aerococcus*, *Clostridia*, *Paludicola*, and *Tuzzerella*) decreased in the gut microbiota of mice treated with Dox (model mice) compared with their abundance in normal mice. The bacterial composition results indicates that the gut microbiota of model mice was a result of the Dox-induced imbalance.

After AVLE treatment, the composition of the gut microbiota of model mice improved to a certain extent. The abundance of nine genera (*Muribaculaceae*, *Ruminococcaceae*, *Eubacterium*, *Roseburia*, *Tyzzerella*, *Staphylococcus*, *Jeotgalicoccus*, *Anaerotruncus*, and *Enterorhabdus*) increased, with a simultaneous decrease in the abundance of 10 genera (*Escherichia−Shigella*, *Akkermansia*, *Bacteroides*, *Proteus*, *Clostridium*, *Ruminococcus*, *Enterococcus*, *Erysipelatoclostridium*, *Enterobacter*, and *Faecalibaculum*). The bacterial composition after AVLE treatment suggests that AVLE has a good effect on regulating gut microbiota. Several genera with increased abundance were reported to be related to the production of IPA and AA in gut microbiota (Wu et al., 2021).

### 3.5 IPA and AA alleviated doxorubicin-induced cardiotoxicity

**FIGURE 5:**
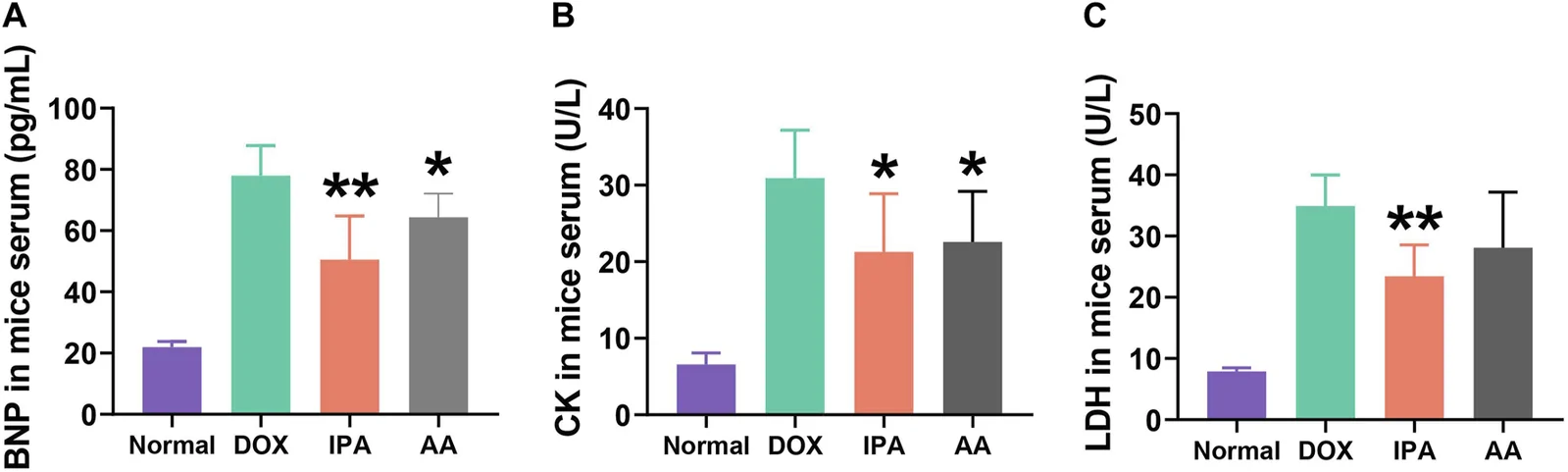
Serum brain natriuretic peptide (BNP), creatine kinase (CK) and lactate dehydrogenase (LDH) of Doxn-induced cardiotoxicity mice after treated with IPA and AA. Data are presented as mean ± SD; n = 6, *p < 0.05, **p < 0.05 compared with model group.

A total of twenty-four C57BL/6 mice were randomly assigned to four groups in order to assess the cardioprotective effects of IPA and AA. Following a 7-week oral administration of either IPA or sodium acetate, the mice’s serum samples were collected, and the levels of BNP, CK, and LDH were measured (Figures 5A–C). The results demonstrated a significant elevation in serum BNP, CK, and LDH levels in the Dox-induced chronic heart failure group, indicative of Dox-induced cardiotoxicity. Conversely, oral administration of IPA or sodium acetate effectively attenuated Dox-induced cardiotoxicity, as evidenced by the reduction in serum BNP, CK, and LDH levels. These findings highlight the cardioprotective potential of AA and IPA.

### 3.6 Cardioprotective function of IPA by inhibiting the apoptosis of cardiomyocyte

**FIGURE 6:**
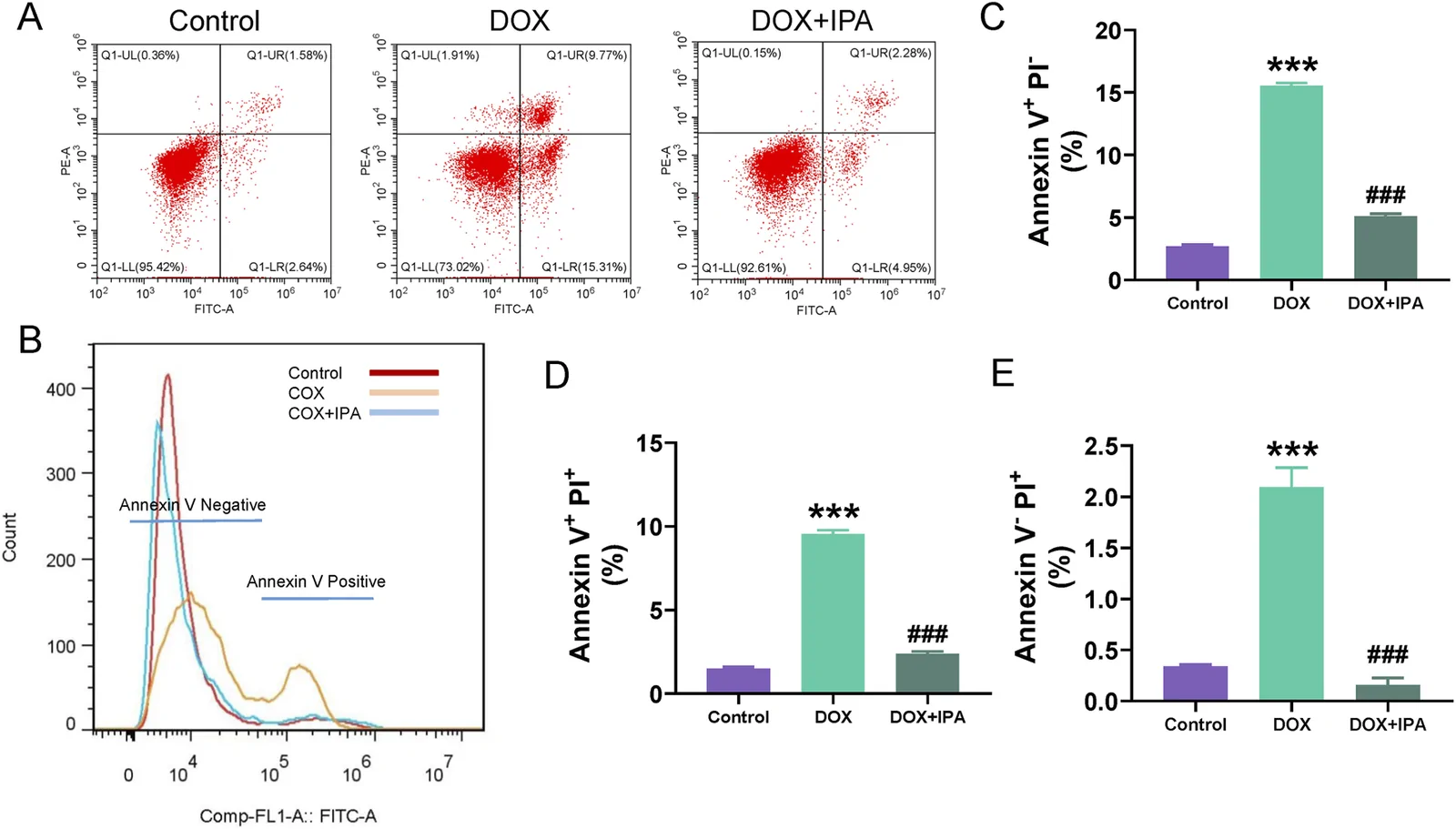
IPA improved cardiomyocyte apoptosis induced by Dox. **(A)** Flow cytometric scatterplot of Annexin-V-FLTC staining of HL-1 cell apoptosis **(B)** Flow cytometric histogram of Annexin-V-FLTC staining of HL-1 cell apoptosis **(C–E)** quantitative analysis of apoptotic HL-1 cells in different apoptosis stages, data are presented as mean ± SD; n = 3, ^###^ *p* < 0.001 compared with Dox group, ***p < 0.001 compared with control group.

Flow cytometry analysis was conducted to investigate the cardioprotective effects of IPA in greater detail. HL-1 cells were pretreated with a concentration of 5 μM IPA for 24 h, followed by treatment with 5 μM Dox for 12 h. As depicted in Figures 6A, B, treatment with Dox alone resulted in a significant increase in apoptosis of HL-1 cells compared to the control group. However, the addition of 5 μM IPA demonstrated a significant inhibition of apoptosis in Dox-treated HL-1 cells. Figures 6C–E illustrate that IPA effectively suppressed both early and late stage apoptosis in HL-1 cells. Collectively, these findings indicate that co-treatment with IPA ameliorates Dox-induced cardiac injury by inhibiting apoptosis.

### 3.7 Cardioprotective function of AA by stimulating secretion of ghrelin (Gh)

**FIGURE 7:**
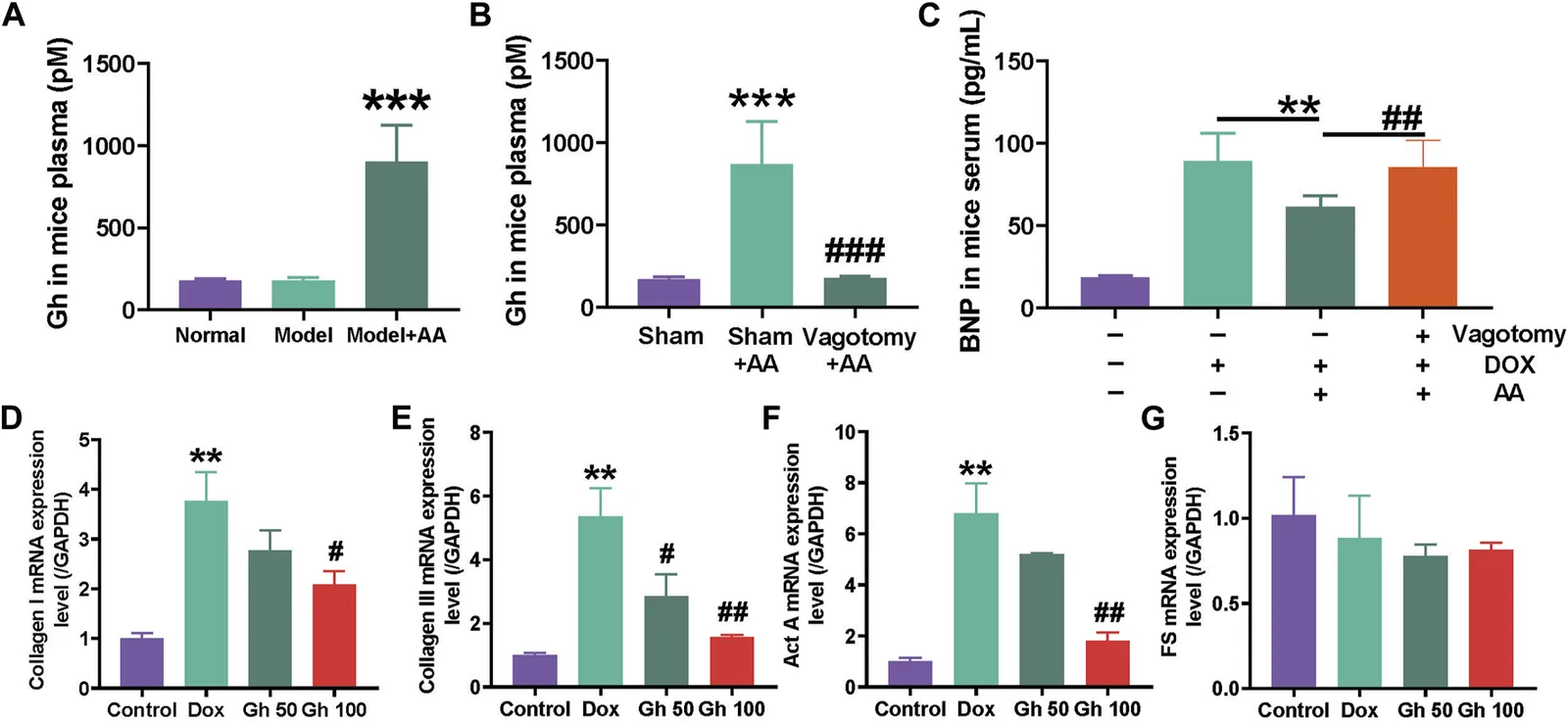
Cardioprotection function of AA by stimulating the secretion of Ghrelin. **(A)** Gh in mice plasma after Dox-induced cardiotoxicity mice treated with AA, data are presented as mean ± SD, n = 6, ***p < 0.001 compared with model group **(B)** Gh in mice plasma after normal mice operated with vagotomy, data are presented as mean ± SD, n = 6, ***p < 0.001 compared with sham group, ^###^ *p* < 0.001 compared with sham AA group **(C)** BNP in mice serum after the 3 days AA treatment, data are presented as mean ± SD, n = 6, **p < 0.01, ^##^ *p* < 0.01 **(D–G)** The mRNA levels of Collagen I, Collagen III, Activin A (Act A) and Follistatin (FS) in HL-1 cell after treated with Gh, data are presented as mean ± SD, n = 3, **p < 0.01 compared with control group, ^#^ *p* < 0.05, ^##^ *p* < 0.01 compared with Dox group.

To further explore the cardioprotective effects of AA, the impact of sodium acetate on Gh secretion was evaluated. Serum Gh levels were measured using an assay kit. The results demonstrated a significant increase in serum Gh levels following sodium acetate treatment (Figure 7A). Notably, the serum Gh levels in normal mice and Dox-treated mice (Model) were comparable at low levels, suggesting that oral AA can stimulate Gh secretion. Vagotomy was performed to disrupt the parasympathetic nerve and investigate the mechanism underlying AA’s effect on Gh. As depicted in Figure 7B, the serum Gh levels of the sham group treated with sodium acetate exhibited a significant increase. Furthermore, the serum Gh levels of vagotomy mice treated with sodium acetate remained at the same level as the sham group, indicating that vagotomy can impede the Gh-stimulating pathway mediated by AA. To further ascertain the cardioprotective effects of AA, vagotomy mice were treated with Dox and AA, and BNP levels were measured to assess cardiac injury. The results (Figure 7C) demonstrated a significant reduction in the cardioprotective effects of AA after vagotomy, suggesting that AA protects the heart by stimulating Gh secretion via the vagus nerve.

Subsequently, the mRNA levels of Act A, FS, collagen I, and collagen III were measured using real-time quantitative PCR in Dox-treated HL-1 cells treated with Gh. The results revealed a significant increase in mRNA levels of collagen I, collagen III, and Act A in Dox-treated HL-1 cells (Figures 7D–F). However, Gh treatment dose-dependently decreased the mRNA levels of collagen I, collagen III, and Act A in Dox-treated HL-1 cells, while the mRNA level of FS remained unchanged.

## 4 Discussion

Doxorubicin (Dox) is commonly employed in the treatment of malignant tumors; however, its clinical use is significantly limited due to the occurrence of acute, sub-acute, or chronic side effects (Rivankar, 2014). Notably, Dox exhibits a high affinity for cardiomyocytes, thereby leading to cardiotoxicity (Wu et al., 2023). The availability of medications for the treatment or prevention of cardiotoxicity is limited. *Apocynum venetum*, known as “Luobuma” in Chinese and “Rafuma” in Japanese, is a perennial herbaceous shrub widely distributed in temperate regions across Asia, Europe, and North America (Xie et al., 2012). Recent studies have demonstrated the beneficial effects of oral A. venetum in mitigating cardiotoxicity with good safety profiles (Zhang et al., 2022). However, the primary bioactive components of A. venetum are flavonoids and polysaccharides, both of which possess low bioavailability (Gong et al., 2020). This limited absorption has hindered research on the cardioprotective mechanism of A. venetum and its subsequent clinical applications. In our study, we established a Dox-induced cardiotoxicity model in C57BL/6 mice to evaluate the cardioprotective effects of AVLE and investigate its underlying mechanism.

To explore the impact of AVLE on the gut microbiota of mice with Dox-induced cardiotoxicity, the analysis focused on organic acids produced by the gut microbiota, which play a significant role in cardiovascular disease, including short-chain fatty acids (SCFAs) and amino acid metabolites. In this study, AVLE treatment was found to significantly increase the levels of indolepropionic acid (IPA) and acetic acid (AA). This increase was further supported by the results of microbial diversity analysis. Specifically, AVLE treatment led to an increased abundance of nine genera, *Muribaculaceae, Ruminococcaceae, Eubacterium, Roseburia, Tyzzerella, Staphylococcus, Jeotgalicoccus, Anaerotruncus*, and *Enterorhabdus*, increased. Among the aforementioned genera, *Muribaculaceae* were enriched in the tryptophan metabolism pathways (Wang et al., 2023); *Enterorhabdus, Ruminococcaceae*, and *Eubacterium* were reported to adjust the co-metabolites, such as SCFAs and IPA (Wu et al., 2021). Therefore, an increase in the abundance of the abovementioned four genera might cause IPA enrichment in the gut microbiota of mice. *Ruminococcaceae* and *Eubacterium* were related to the metabolism of SCFAs (Chen et al., 2019), especially acetate metabolism, and the abundance of *Anaerotruncus* showed a positive correlation with faecal acetic acid concentrations after piglets were treated with *L. plantarum* (Wang et al., 2018). The above evidence indicates that the increased abundance of *Ruminococcaceae*, *Eubacterium*, and *Anaerotruncus* could increase AA level in the gut microbiota of mice.

Subsequently, we investigated the cardioprotective effects of two organic acids regulated by AVLE, namely, indolepropionic acid (IPA) and acetic acid (AA). Flow cytometry analysis was employed to examine the cardioprotective functions of IPA. As depicted in Figure 6, IPA demonstrated the ability to inhibit apoptosis in cardiomyocytes at both early and late stages. Dox has been known to induce mitochondrial oxidative stress, disrupt mitochondrial oxidative phosphorylation, and cause permeability transition, leading to perturbations in metabolic and redox circuits within cardiac cells. These alterations ultimately result in dysregulation of autophagy/mitophagy processes and increased apoptosis (Wallace et al., 2020). Additionally, previous studies have reported that IPA can dose-dependently enhance cardiac contractility by modulating mitochondrial function in cardiomyocytes (Gesper et al., 2021). In conclusion, IPA may exert its inhibitory effects on cardiomyocyte apoptosis by mitigating doxorubicin-induced mitochondrial injury.

**FIGURE 8:**
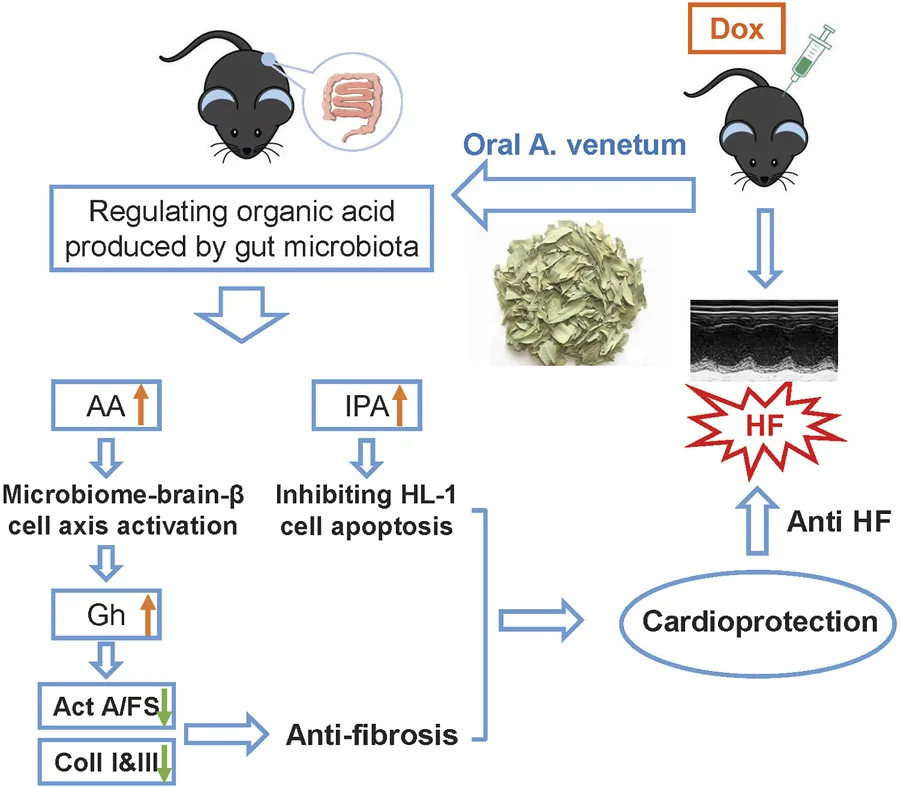
Potential mechanism of AVLE treated Dox-induced cardiotoxicity by regulating gut microbita.

 In addition to IPA, AA emerged as another active organic acid regulated by AVLE in the gut microbiota of mice. Oral administration of sodium acetate, a source of AA, resulted in decreased levels of serum B-type natriuretic peptide (BNP), creatine kinase (CK), and lactate dehydrogenase (LDH) in mice with Dox-induced cardiotoxicity, indicating the beneficial cardioprotective effects of AA (Perry et al., 2016). Subsequently, Gh could activate the Akt-mTOR signalling pathway by combining with hormone secretagogue receptor (Wang et al., 2017) and adjust Act A/FS imbalance to inhibit myocardial fibrosis, which further enhanced the viability of the cardiac muscle cells and inhibited the cell apoptosis, thereby protecting the cardiomyocytes from injury (Yang et al., 2018). In this study, to investigate the role of Gh-stimulated AA in cardioprotection, vagotomy was performed to block Gh stimulation, resulting in the loss of AA’s cardioprotective effect in mice with Dox-induced cardiotoxicity. Subsequently, real-time quantitative PCR was conducted to measure the mRNA levels of Act A, follistatin, collagen I, and collagen III in HL-1 cells treated with Dox and Gh. The mRNA levels of collagen I and collagen III serve as markers for myocardial fibrosis development and can even predict the prognosis of heart failure. Act A, a member of the transforming growth factor-β superfamily of cytokines, plays a crucial role in inflammation, immunity, and fibrosis, while follistatin acts as a binding protein that can antagonize the action of Act A (Yang et al., 2018; Nikolov and Popovski, 2022). Therefore, maintaining a balance between Act A and follistatin is closely associated with the pathological process of myocardial fibrosis (Bloise et al., 2019). In this study, we observed an imbalance in the Act A/FS system following Dox treatment in HL-1 cells, primarily attributed to increased Act A expression. Gh administration effectively ameliorated this imbalance by reducing Act A levels, suggesting its potential to suppress cardiac fibrosis. Overall, AA promotes increased Gh secretion through the parasympathetic nervous system, and Gh, in turn, suppresses cardiac fibrosis by decreasing levels of collagen I, collagen III, and Act A (Figure 8).

Figure 8 provides a schematic representation of the mechanism underlying the cardioprotective effect of AVLE, highlighting the significant impact of AVLE on the regulation of gut microbiota. Following AVLE stimulation, the levels of two organic acids produced by the gut microbiota, namely, IPA and AA, are increased. It is proposed that IPA may exert its cardioprotective effect by mitigating Dox-induced mitochondrial injury, thereby inhibiting cardiomyocyte apoptosis. On the other hand, AA facilitates the secretion of Gh through the parasympathetic nervous system, leading to the suppression of cardiac fibrosis by reducing the levels of collagen I, collagen III, and Act A. While the modulation of gut microbiota by AVLE does not represent the complete pathway underlying its cardioprotective effect, it underscores the undeniable significance of targeting the gut microbiota for the treatment of cardiac diseases.

## 5 Conclusion

In summary, our study demonstrates the potential of oral AVLE as a treatment or preventive measure for Dox-induced cardiotoxicity. AVLE exerts its effects through the regulation of gut microbiota, leading to the generation of IPA and AA. It is suggested that IPA may mitigate cardiomyocyte apoptosis by alleviating Dox-induced mitochondrial injury. Additionally, AA promotes increased Gh secretion, which in turn suppresses cardiac fibrosis. Our findings highlight AVLE as a promising dietary supplement for the prevention of Dox-induced cardiotoxicity, and further identify IPA and AA as potential therapeutic targets for cardiac diseases.

## Data Availability

The datasets presented in this study can be found in online repositories. The names of the repository/repositories and accession number(s) can be found below: https://www.scidb.cn/s/y2iIBn, DOI: 10.57760/sciencedb.09973.
