# Correction: *Apocynum venetum* leaf extract alleviated doxorubicin-induced cardiotoxicity by regulating organic acid metabolism in gut microbiota

**DOI:** 10.3389/fphar.2026.1966401

**Published:** 2026-09-03

**Authors:** Zhenxiong Zhao, Shenglu Jiang, Qing Fan, Kuo Xu, Yubin Xu, Feiqiang Wu, Xihong Zhang, Ting Wang, Zhelin Xia

**Affiliations:** 1 Taizhou Central Hospital (Taizhou University Hospital), Taizhou, Zhejiang, China; 2 School of Medicine, Taizhou University, Taizhou, Zhejiang, China; 3 Shandong Cancer Hospital and Institute, Shandong First Medical University and Shandong Academy of Medical Sciences, Jinan, China; 4 Marine Traditional Chinese Medicine Research Center, Shandong University of Traditional Chinese Medicine, Jinan, China; 5 Taizhou Hospital of Zhejiang Province Affiliated to Wenzhou Medical University, Taizhou, Zhejiang, China

**Keywords:** gut microbiota, *Apocynum venetum* leaf, doxorubicin, cardiotoxicity, indole-3-propionic acid

There was a mistake in Figure 6 as published, concerning the Y-axis label of Figures 6C,D and E. The Y-axis label of Figures 6C,D and E was incorrectly annotated during the preparation of the original figure. The corrected Figure 6 appears below.

**FIGURE 6:**
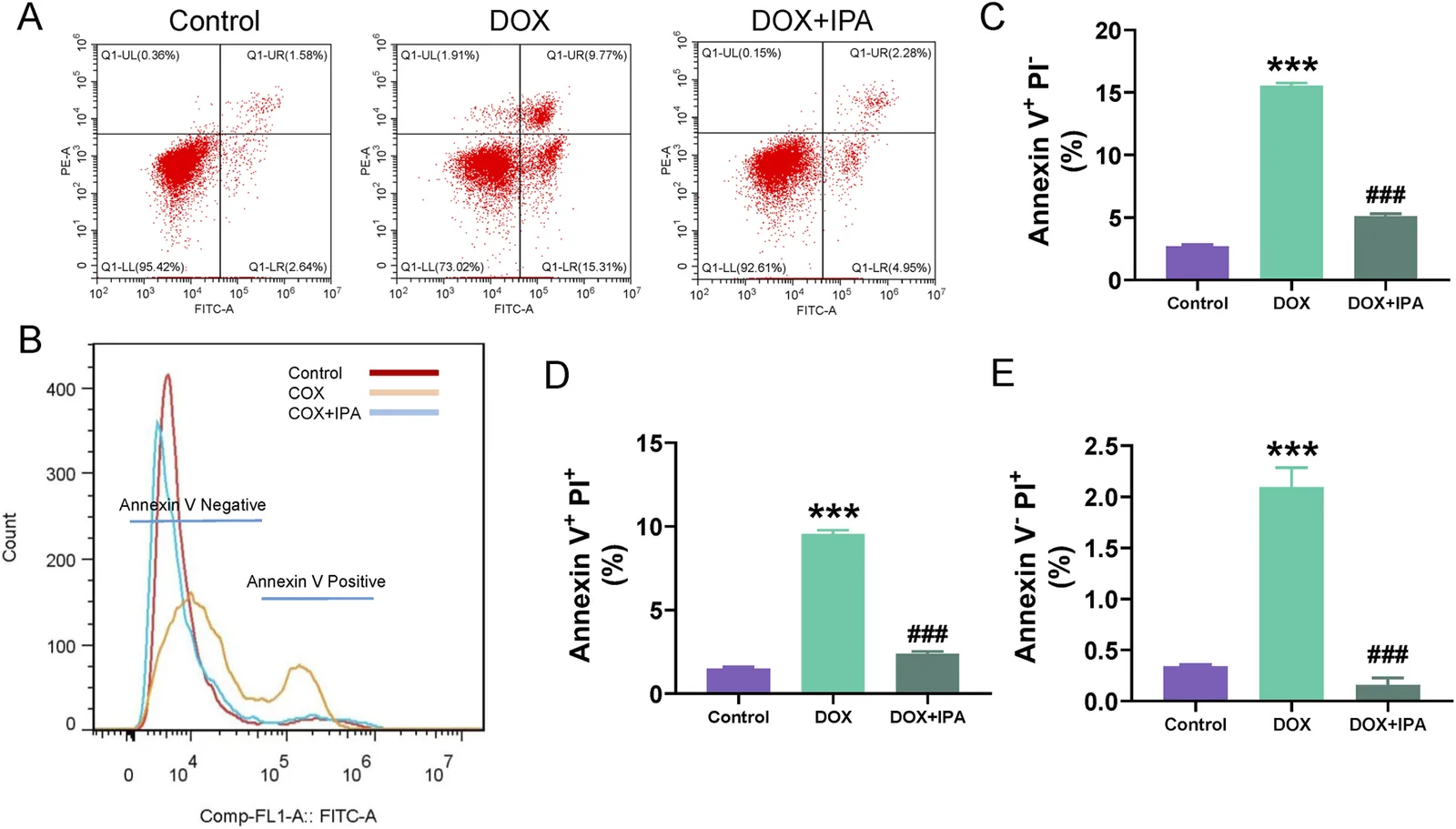
IPA improved cardiomyocyte apoptosis induced by Dox. **(A)** Flow cytometric scatterplot of Annexin-V-FLTC staining of HL-1cell apoptosis **(B)** Flow cytometric histogram of Annexin-V-FLTC staining of HL-1 cell apoptosis **(C–E)** quantitative analysis of apoptotic HL-1cells in different apoptosis stages, data are presented as mean ± SD; n = 3, ^###^ *p* < 0.001 compared with Dox group, ^***^ *p* < 0.001 compared with control group.

The original version of this article has been updated.

