# Supplementary material for: Apocynum venetum leaf extract alleviated doxorubicin-induced cardiotoxicity by regulating organic acid metabolism in gut microbiota
Source: Front Pharmacol. 2023 Nov 23;14:1286210. doi: 10.3389/fphar.2023.1286210 (PMC10701262; doi:10.3389/fphar.2023.1286210)
Supplement: Supplementary file 1 [file DataSheet2.docx]

Supplemental Material-2

Method 1:

The analysis was performed using Shimadzu HPLC system (Kyoto, Japan) LC-2040 and Kromasil 100-5 C_18_ column (4.6 mm×250 mm, 5 μm, AkzoNobel Global) with a flow rate of 0.5 mL/min. The mobile phase consisted of formic acid:water (0.15:100, v/v) (as phase A) and acetonitrile:isopropanol (95:5, v/v) (as phase B), with a binary gradient elution as follows (B): 0 min: 12%, 15 min: 13%, 25 min: 17%, 50 min: 18%, 65 min: 21%, 80 min: 80%. The column temperature was set as follows: 0 min: 35^o^C, 40 min: 50^o^C, 80 min 50^o^C. The flow rate was 0.5 mL/min, and the detection wavelength was 360 nm.

Figure 1 The chromatogram ofmixed reference substance using method 1. Hyperoside (1 μg/mL) and isoquercetin (4μg/mL).

Figure 2 The chromatogram of AVLE using method 1.

Method 2:

The analysis was performed using Shimadzu HPLC system (Kyoto, Japan) LC-2040 and Kromasil 100-5 C_18_ column (4.6 mm×250 mm, 5 μm, AkzoNobel Global) with a flow rate of 0.5 mL/min. The mobile phase consisted of formic acid:water (0.15:100, v/v) (as phase A) and acetonitrile:isopropanol (95:5, v/v) (as phase B), with a binary gradient elution as follows (B): 0 min: 12%, 15 min: 13%, 25 min: 17%, 50 min: 18%, 65 min: 21%, 80 min: 80%. The column temperature was set as follows: 0 min: 35^o^C, 40 min:50^o^C, 80 min 50^o^C. The flow rate was 0.5 mL/min, and the detection wavelength was 254 nm.

Figure 3 The chromatogram ofmixed reference substance using method 2. Hyperoside (1 μg/mL) and isoquercetin (4μg/mL).

Figure 4 The chromatogram of AVLE using method 2.

Method 3:

The analysis was performed using Shimadzu HPLC system (Kyoto, Japan) LC-2040 and Kromasil 100-5 C_18_ column (4.6 mm×250 mm, 5 μm, AkzoNobel Global) with a flow rate of 0.5 mL/min. The mobile phase consisted of formic acid:water (0.2:100, v/v) (as phase A) and acetonitrile:isopropanol (95:5, v/v) (as phase B), with a binary gradient elution as follows (B): 0 min: 12%, 15 min: 13%, 25 min: 17%, 50 min: 18%, 65 min: 21%, 80 min: 80%. The column temperature was set as follows: 0 min: 35^o^C, 40 min: 50^o^C, 80 min 50^o^C. The flow rate was 0.6 mL/min, and the detection wavelength was 360 nm.

Figure 5 The chromatogram of mixed reference substance using method 3. Hyperoside (1 μg/mL) and isoquercetin (4 μg/mL).

Figure 6 The chromatogram of AVLE using method 3.

Method 4:

The analysis was performed using Shimadzu HPLC system (Kyoto, Japan) LC-2040 and ZORBAX SB-C_18_ column (4.6 mm×250 mm, 5 μm, Agilent) with a flow rate of 0.5 mL/min. The mobile phase consisted of formic acid:water (0.15:100, v/v) (as phase A) and acetonitrile:isopropanol (95:5, v/v) (as phase B), with a binary gradient elution as follows (B): 0 min: 12%, 15 min: 13%, 25 min: 17%, 50 min: 18%, 65 min: 21%, 80 min: 80%. The column temperature was set as follows: 0 min: 35^o^C, 40 min:50^o^C, 80 min 50^o^C. The flow rate was 0.5 mL/min, and the detection wavelength was 360 nm.

Figure 7 The chromatogram of mixed reference substance using method 4. Hyperoside (1 μg/mL) and isoquercetin (4 μg/mL).

Figure 8 The chromatogram of AVLE using method 4.

Table 1 Determination of main active ingredients using three different methods

|  | Hyperoside | | Isoquercetin | |
| --- | --- | --- | --- | --- |
|  | Concentration (μg/mL) | Content (%) | Concentration (μg/mL) | Content (%) |
| Method 1 | 19.52 | 0.98 | 58.25 | 2.91 |
| Method 2 | 17.68 | 0.88 | 59.33 | 2.97 |
| Method 3 | 24.13 | 1.21 | 61.88 | 3.09 |
| Method 4 | 22.88 | 1.14 | 62.93 | 3.15 |
