# Supplementary material for: Apocynum venetum leaf extract alleviated doxorubicin-induced cardiotoxicity by regulating organic acid metabolism in gut microbiota
Source: Front Pharmacol. 2023 Nov 23;14:1286210. doi: 10.3389/fphar.2023.1286210 (PMC10701262; doi:10.3389/fphar.2023.1286210)
Supplement: Supplementary file 2 [file DataSheet1.docx]

Supplemental Material-1

Figure S1 Mass spectrometry TIC of AVLE


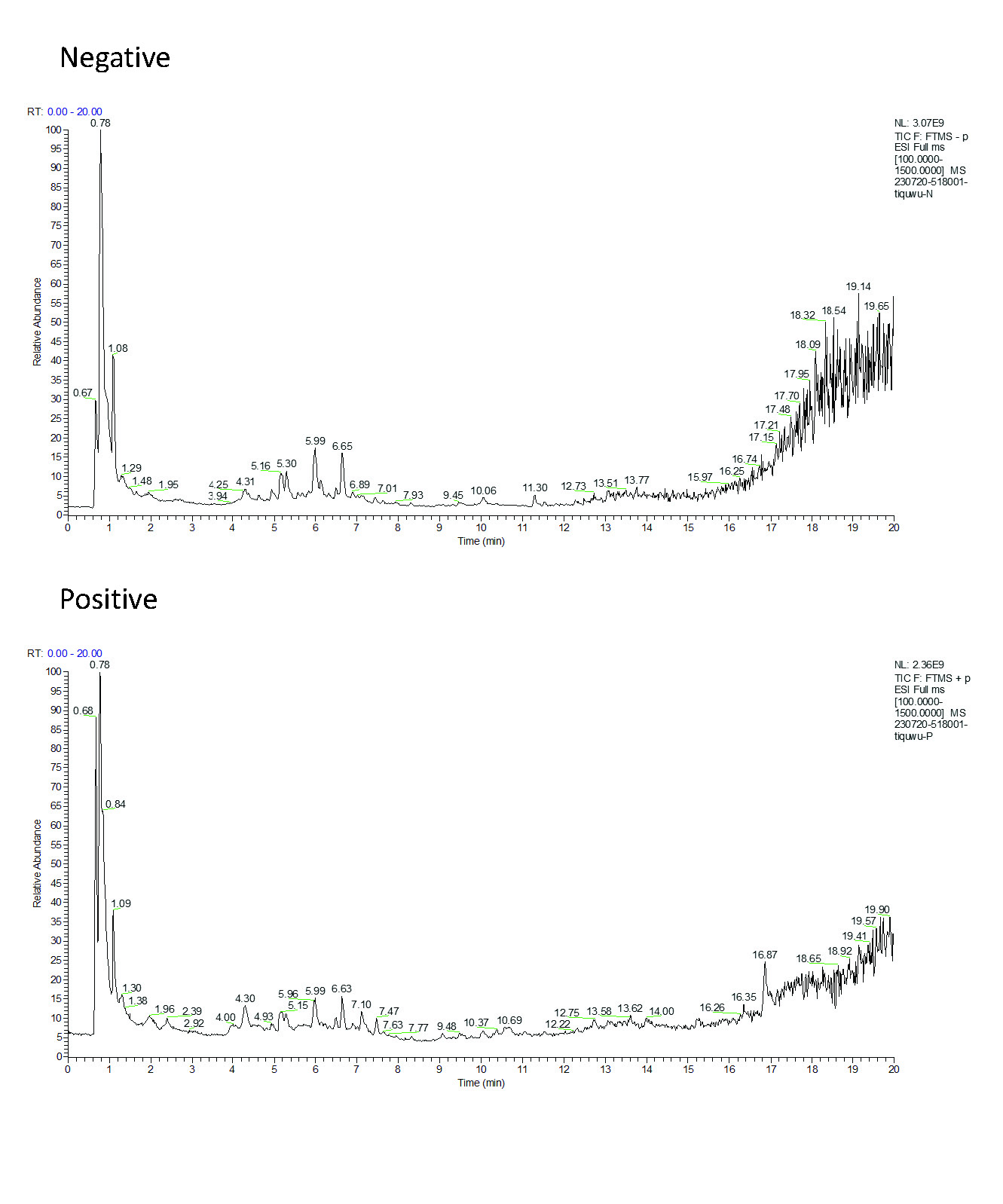


Figure S2 Microbial diversity of mice gut microbiota. (A) Beta diversity Anosim test, N (normal group), M (model group), A (A2 group); (B) The relative abundance of the gut microbiota genus in each sample, N (normal group), M (model group), A (A2 group).


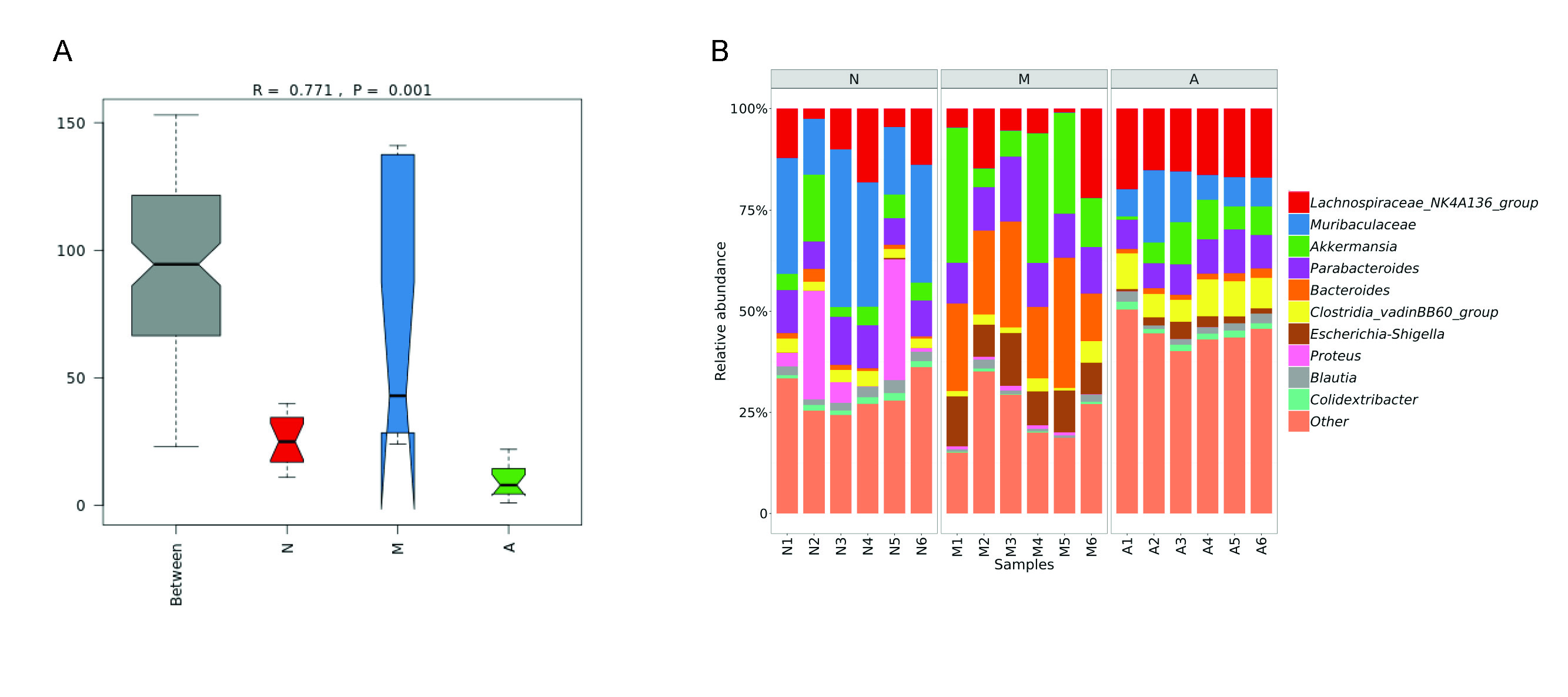


Table S1 Primer sequences used in real-time quantitative PCR

| Target | Genbank | Primer | Sequences |
| --- | --- | --- | --- |
| Activin A | NM_001110204.1 | Sense | CAGGAGAGTGTTTCCTCGTCC |
|  |  | Antisense | ATGGGCTTCCCGTTGATGAC |
| Follistatin | NM_001301373.1 | Sense | GCTGCATAGCAGATTTGGGC |
|  |  | Antisense | CACAGAACAAGGCCAAAGGC |
| Collagen I | NM_007742.4 | Sense | ATGCCCACAGCCTTCTACAC |
|  |  | Antisense | CGGATAGCCACCCATTCCTC |
| Collagen III | NM_009930.2 | Sense | AAGATATCCAGTGTGGCGGC |
|  |  | Antisense | GTCCTGGTCTTCCTCCTCCT |
| Gapdh | X02231.1 | Sense | CATCACCATCTTCCAGGAGCG |
|  |  | Antisense | TGACCTTGCCCACAGCCTTG |

Table S2 Main components in AVLE

| No. | Compound | Formula | Annotation MW | Error (ppm) | Content (%) |
| --- | --- | --- | --- | --- | --- |
| 1 | 9,12-Octadecadienal | C_18_H_32_O | 264.24532 | -3.68 | 11.299 |
| 2 | Hyperoside | C_21_H_20_O_12_ | 464.09548 | -1.39 | 8.866 |
| 3 | 5-Hydroxymethylfurfural | C_6_H_6_O_3_ | 126.03169 | -0.95 | 5.770 |
| 4 | Cryptochlorogenic acid | C_16_H_18_O_9_ | 354.09508 | -1.61 | 5.530 |
| 5 | Linoleic acid | C_18_H_32_O_2_ | 280.24023 | -2.32 | 5.513 |
| 6 | 1-Caffeoylquinic acid | C_16_H_18_O_9_ | 354.09508 | -1.9 | 3.887 |
| 7 | Chlorogenic acid | C_16_H_18_O_9_ | 354.09508 | -1.77 | 3.799 |
| 8 | Isoquercitrin | C_21_H_20_O_12_ | 464.09548 | -2.11 | 3.332 |
| 9 | D-Sucrose | C_12_H_22_O_11_ | 342.11621 | -3.28 | 3.317 |
| 10 | Caffeic acid | C_9_H_8_O_4_ | 180.04226 | -1.91 | 1.679 |
| 11 | Quercetin 3,7-diglucoside | C_27_H_30_O_17_ | 626.1483 | -2.04 | 1.614 |
| 12 | Betaine | C_5_H_11_NO_2_ | 117.07898 | -0.6 | 1.576 |
| 13 | Dianthoside | C_12_H_16_O_8_ | 288.08452 | -2.76 | 1.422 |
| 14 | Paeoniflorin | C_23_H_28_O_11_ | 480.16316 | -1.87 | 1.364 |
| 15 | Morin | C_15_H_10_O_7_ | 302.04265 | -2.74 | 1.200 |
| 16 | benzoquinone | C_6_H_4_O_2_ | 108.02113 | 0.22 | 1.098 |
| 17 | Adenosine | C_10_H_13_N_5_O_4_ | 267.09675 | -2.96 | 1.033 |
| 18 | 8,9-Dihydroxy-9-(2-methyl-2-propanyl)dihydro-9H-furo[2,3-b]furo[3',2':2,3]cyclopenta[1,2-c]furan-2,4,7(3H,8H)-trione | C_15_H_18_O_8_ | 326.10017 | -2.1 | 0.873 |
| 19 | Lariciresinol 4-O-glucoside | C_26_H_34_O_11_ | 522.21011 | -1.66 | 0.798 |
| 20 | Astragalin | C_21_H_20_O_11_ | 448.10056 | -1.41 | 0.776 |

The methods of H&E and Masson staining

The pre-prepared paraffin sections were de-paraffinized with xylene (I) and (II) respectively for 5 min, then treated with a gradient of ethanol (anhydrous ethanol for 5 min, 95% ethanol for 2 min, 80% ethanol for 2 min, 70% ethanol for 2 min), followed by distilled water for 2 min. The de-paraffinized tissue section was dyed with hematoxylin dyeing solution for 20 min, and then washed with tap water. Next, differentiation solution was applied for 30 s and the tissue samples were soaked in water for 15 min. Then samples were stained with eosin dyeing solution for 30 s and rinsed under running water. After the samples were immersed in water for 5 min, ethanol gradient dehydration was performed and followed by xylene transparency and sealed with neutral gum. Finally, an optical microscope was used to observe and photograph.

Masson staining procedure was as following: first, the paraffin sections were dewaxed using xylene, and then dyed with Weigert iron hematoxylin staining solution for 5 min. The acidic ethanol differentiation solution was used for differentiation for 5 s, and washed with water. Masson blue solution was applied to return to blue for 3 min and then the samples were rinsed with distilled water for 1 min. After this, the samples were stained with Lichunhong Magenta staining solution for 5 min, and then washed with a weak acid working solution (mixed solution of distilled water and weak acid solution with a ratio of 2:1) for 1 min, phosphomolybdic acid solution for 2 min and weak acid working solution again for 1 min. Samples were then stained with aniline blue staining solution for 2 min, and washed with weak acid working solution for 1 min. Then samples were dehydrated with 95% ethanol rapidly and with absolute ethanol for 3 times. After xylene transparency for 3 times, the samples were sealed with neutral gum. Finally, an optical microscope was used to observe and photograph.

The methods of Microbial diversity analysis

The samples were freezed under -80 oC. The V3-V4 region of the bacteria’s 16S rRNA gene was amplified with primers 338F and 806R. AxyPrep DNA GelExtraction Kit (Axygen Biosciences, UnionCity,CA,USA)was used to purify the amplicons, followed by quantification using QuantiFluor-ST (Promega, Madison, WI, USA). Illumina MiSeq instrument (Illumina, San Diego, CA, USA) was used for sequencing. The 16S rRNA sequencing data was analyzed with Quantitative Insights Into Microbial Ecology platform (V.1.9.1). Operational taxonomic units (OTUs) with similarity over 97% were selected for taxonomy identification with Greengenes database (V.13.8).
